# Using Generalized Anxiety Disorder-2 (GAD-2) and GAD-7 in a Primary Care Setting

**DOI:** 10.7759/cureus.8224

**Published:** 2020-05-21

**Authors:** Amit Sapra, Priyanka Bhandari, Shivani Sharma, Trupesh Chanpura, Lauri Lopp

**Affiliations:** 1 Family Medicine, Southern Illinois University School of Medicine, Springfield, USA; 2 Internal Medicine, Springfield Clinic, Springfield, USA

**Keywords:** generalized anxiety disorder, primary care, generalized anxiety disorder questionnaire, anxiety, mental health services, mental health, mental health screening, general & adult psychiatry

## Abstract

Anxiety disorders are highly prevalent in the primary care setting and are responsible for significant morbidity as well as a loss of productivity. Evaluation by mental health specialists and behavioral specialists can sometimes be confounded with problems of availability, accessibility, and the patient’s hesitancy to talk to new providers due to lack of prior relationship and trust. Primary care providers already have the advantage of being available for their patients, and have built years of trust behind them to strengthen this relationship. The biggest problems which confront primary care are the time constraints as well as the presence of multiple medical demands. This leads to a constant need for tools that facilitate early recognition and diagnosis of mental health disorders while also providing judicious utilization of clinic time. This article attempts to review the use of two of these popular tools: Generalized Anxiety Disorder scale-2 (GAD-2) and GAD-7 in the primary care setting.

## Introduction and background

Anxiety is a normal human emotion, but sometimes it can become excessive, and pervasive, and instead assumes pathological significance, and becomes a disorder. At this stage, the anxiety disorder potentially starts affecting the instrumental activities of daily living (IADL), which comprise activities like driving, cleaning, managing finances [1]. When these instrumental activities of daily living spiral down, personal and professional aspects of life decline. 

Anxiety also presents with comorbidities like depression, phobias, post-traumatic stress disorder, alcohol, and drug dependence. These comorbidities interfere with life’s activities and increase the risk of chronicity, hospitalizations, and even suicide attempts [2]. Primary care providers who are working diligently to provide care for their patients on issues of competing medical importance, within the limited time constraints, can find diagnosing these anxiety disorders quite challenging. Undoubtedly, this has created a need for screening tools that are efficient, reliable, comprehensible, easy to administer, and reproducible.

The Generalized Anxiety Disorder scale-7 (GAD-7) is a seven-item diagnostic tool validated in both the primary care setting and the general population [3]. The GAD-2 is an ultra-quick version of the seven-item scale that incorporates the first two questions of the GAD-7, which are also critical components of any anxiety disorder.

## Review

Generalized anxiety disorder (GAD) is very prevalent in the general population, with a current prevalence of 2% to 3% and a lifetime prevalence of over 5% [3,4]. It is also the most prevalent anxiety disorder encountered in primary care, with an estimated point prevalence of 8% [5]. In a study in Finland, among primary care patients, its prevalence was reported to be 4.1% for males and 7.1% for females [6]. The rates of anxiety disorders are twice as prevailing in the female population as compared to their male counterparts. It has been consistently found that the prevalence of anxiety disorders in women is probably attributed to genetic and neurobiological causes [7].

Patients with GAD can often present with somatic symptoms, which can cause a diagnostic predicament [8]. Hence, it is not surprising that health care professionals poorly detect GAD at the primary care level [8]. GAD shows higher comorbidity with other mental health disorders like depression and somatization [9].

Multiple scales have been developed for assessing the presence of anxiety that has been validated in the clinical settings. These include the Hamilton Anxiety Scale (HAM-A), Hospital Anxiety and Depression Scale (HADS), Covi Anxiety Scale, Clinical Anxiety Scale (CAS), State-Trait Anxiety Inventory (STAI), Generalized Anxiety Disorder Questionnaire-IV (GAD-Q-IV), and World Health Organization Composite International Diagnostic Interview Short-Form (CIDI-SF), among others [10-16].

The GAD-7 is a recently developed easy to use 7-item scale, based on Diagnostic and Statistical Manual of Mental Disorders-IV (DSM-IV) criteria, for identifying likely cases of GAD (Table 1). It has been found to have great psychometric properties and is short and easy to administer. This allows the GAD-7 to be used in remote health surveys, epidemiologic studies, and also in primary care settings [17].

**Table 1:** The GAD-7 Scale [18]

Generalized Anxiety Disorder 7- item (GAD-7)				
Over the last 2 weeks, how often have you been bothered by the following problems	Not at all	Several days	More than half the days	Nearly every day
1. Feeling nervous, anxious or on edge	0	1	2	3
2. Not being able to stop or control worrying	0	1	2	3
3. Worrying too much about different things	0	1	2	3
4. Trouble relaxing	0	1	2	3
5. Being so restless that it is hard to sit still	0	1	2	3
6. Becoming easily annoyed or irritable	0	1	2	3
7. Feeling afraid as if something awful might happen	0	1	2	3
GAD-7 score obtained by adding score for each question (total points).				
A score of 8 points or higher is a reasonable is the cut-off for needing further identifying evaluation to determine presence and type of anxiety disorder ^23, 24^				
The following cut-offs correlate with level of anxiety severity:				
Score 0-4	: Minimal Anxiety			
Score 5-9	: Mild Anxiety			
Score 10-14:	: Moderate Anxiety			
Score 15 or greater	: Severe Anxiety			

As per the review of literature from Archives of Internal Medicine, 'A Criterion' -a standard study was conducted between November 2004 and June, 2005 in 15 primary care clinics with around 3000 patients in the United States [18]. The GAD-7 self-reported scale was compared with an independent diagnosis made by mental health professionals. The project was carried in two phases with the emphasis in the first phase being on selecting the scale items and the cut off scores. The second phase emphasized on testing the reliability of the scale.

The GAD-7 is a 7-item scale that has reporting scores from 0 to 3 on all the questions (Table 1). It investigates how often the patient has been bothered by seven different symptoms of anxiety during the last two weeks with response options such as: “ not at all,” “ several days ‘,“ more than half the days,” and “ nearly daily ” scored as 0, 1, 2, and 3, respectively. The scores of 5, 10, and 15 are taken as cut off points for mild, moderate, and severe anxiety, respectively. If we use a threshold of 10, then the GAD-7 assumes an excellent sensitivity of 89% and a specificity of 82% for GAD [18]. It is thus a potent and efficient tool for screening GAD. It also performs moderately well at detecting three other common anxiety disorders, including panic disorder (sensitivity 74%, specificity 81%), social anxiety disorder (sensitivity 72%, specificity 80%), and post-traumatic stress disorder (sensitivity 66%, specificity 81%) [18].

The initial scale was developed as a 13-item scale but was subsequently moved to the 7-item scale called the GAD-7 [18]. The 7-item questionnaire showed highest correlation with the total 13-item scale score (r = 0.75-0.85) [18]. The ROC with this set of items showed an area under the curve (0.906), which was found to be as good as the scales with the full 13-item set. The two core criteria (A and B) of the DSM-IV definition of generalized anxiety disorder are well captured by the first three items of the questionnaire [18]. GAD-7 scale was found to have excellent internal consistency (Cronbach α = .92) as well as a good test-retest reliability (intraclass correlation = 0.83) (Table 2) [18].

The GAD-7 Scale has been validated within a large sample of patients in a primary care setting in multiple studies and across numerous nations [1,19-23].

**Table 2:** Performance of GAD-7 as Screening Tool for Anxiety Disorders [23]

Performance of GAD-7 as Screening Tool for Anxiety Disorders (Using GAD-2 Score Cut-off of ≥3)			
Anxiety Disorder	Sensitivity	Specificity	Positive Likelihood Ratio
Generalized Anxiety Disorder	89%	82%	5.1
Panic Disorder	74%	81%	3.9
Social Anxiety Disorder	72%	80%	3.6
Post-Traumatic Stress Disorder	66%	81%	3.5
Any Anxiety Disorder	68%	88%	5.5

There had been multiple suggestions to shorten the questionnaire further and use only the first two questions of the GAD-7, which relate to the two main problems of generalized anxiety disorder.

This led to the development of GAD-2, which is the shorter version of GAD-7 and uses only the first two questions, which represent the core anxiety symptoms (Tables 3-4). It tries to highlight the important components which are present regardless of the underlying specific diagnosis.

Studies done by Plummer et al. have reported a good sensitivity of 76% and specificity of 81% with the GAD-2 [24].

**Table 3:** The GAD-2 Scale [18]

Generalized Anxiety Disorder 2 item (GAD-2)				
Over the last 2 weeks, how often have you been bothered by the following problems	Not at all	Several days	More than half the days	Nearly every day
1. Feeling nervous, anxious or on edge	0	1	2	3
2. Not being able to stop or control worrying	0	1	2	3
GAD-2 score obtained by adding score for each question (total points).				
A score of 3 points is the preferred cut-off for needing further identifying evaluation ^23^				

**Table 4:** Performance of GAD-2 Scale as a screening tool for Anxiety Disorders [25]

Performance of GAD-2 as Screening Tool for Anxiety Disorders(Using GAD-2 Score Cut-off of ≥3)			
Anxiety Disorder	Sensitivity	Specificity	Positive Likelihood Ratio
Generalized Anxiety Disorder	86%	83%	5.0
Panic Disorder	76%	81%	4.1
Social Anxiety Disorder	70%	81%	3.6
Post-Traumatic Stress Disorder	59%	81%	3.1
Any Anxiety Disorder	65%	88%	5.2

The GAD-2 questionnaire has been validated in multiple studies and shown to retain the excellent psychometric properties of the GAD-7. Due to its discriminant capability, it has been proposed as an essential first step for screening generalized anxiety disorder [24]. In primary care clinical encounters where time is a constraint, the provider can resort to using the GAD-2 and follow up with the patient for further evaluation [24].

## Conclusions

Anxiety disorders are a prevalent entity in the primary care setting, accounting for a significant decrease in quality of life as well as loss in productivity. The GAD-2 and GAD-7 questionnaires are quick to administer, enhancing time efficiency. Both questionnaires also maintain good sensitivity and specificity for the diagnosis of the most common anxiety disorders encountered in primary care.
